# Routine invasive management after fibrinolysis in patients with ST-elevation myocardial infarction: a systematic review of randomized clinical trials

**DOI:** 10.1186/1471-2261-11-34

**Published:** 2011-06-20

**Authors:** Peter Bogaty, Kristian B Filion, James M Brophy

**Affiliations:** 1 Institut universitaire de cardiologie et pneumologie de Québec, Quebec, Canada; 2 Division of Epidemiology and Community Health School of Public Health University of Minnesota, Minneapolis, MN, USA; 3 McGill University Health Center, McGill University, Montreal, Quebec, Canada

## Abstract

**Background:**

Patients with ST-elevation myocardial infarction (STEMI) treated with fibrinolysis are increasingly, and ever earlier, referred for routine coronary angiography and where feasible, undergo percutaneous coronary intervention (PCI). We sought to examine the randomized clinical trials (RCTs) on which this approach is based.

**Methods:**

We systematically searched EMBASE, Medline, and references of relevant studies. All contemporary RCTs (published since 1995) that compared systematic invasive management of STEMI patients after fibrinolysis with standard care were included. Relevant study design and clinical outcome data were extracted.

**Results:**

Nine RCTs that randomized a total of 3320 patients were identified. All suggested a benefit from routine early invasive management. They were individually reviewed but important design variations precluded a formal quantitative meta-analysis. Importantly, several trials did not compare a routine practice of invasive management after fibrinolysis with a more selective 'ischemia-guided' approach but rather compared an early versus later routine invasive strategy. In the other studies, recourse to subsequent invasive management in the usual care group varied widely. Comparison of the effectiveness of a routine invasive approach to usual care was also limited by asymmetric use of a second anti-platelet agent, differing enzyme definitions of reinfarction occurring spontaneously versus as a complication of PCI, a preponderance of the 'soft' outcome of recurrent ischemia in the combined primary endpoint, and an interpretative bias when invasive procedures on follow-up were tallied as an endpoint without considering initial invasive procedures performed in the routine invasive arm.

**Conclusions:**

Due to important methodological limitations, definitive RCT evidence in favor of routine invasive management following fibrinolysis in patients with STEMI is presently lacking.

## Background

### The Case

A 66-year-old man presented at 4 AM at a community hospital with chest pain of 90 min duration. His vital signs were stable. The ECG showed ST-segment elevation of 3-5 mm in inferior leads and precordial leads V5-V6 and ST-segment depression of 3-4 mm in leads V1-V3. He received aspirin, clopidogrel, intravenous morphine and bolus fibrinolytic therapy 2 h after the start of his symptoms followed by low molecular weight heparin. Over the next hour, the pain subsided significantly and an ECG showed over 50% resolution of ST-elevation. He was awakened at 8 AM for an ECG that showed Q-waves and 1 mm ST-elevation and T-wave inversion in inferior leads and T-wave inversion only in V5-V6. Should the patient be transferred to the nearest tertiary cardiac center 550 km away for cardiac catheterization and revascularization or progressively mobilized and risk stratified with an ECG stress test a few days later?

### The Case Revisited

The treating clinicians believed the patient should be transferred to the tertiary cardiac center for coronary angiography and revascularization if feasible. However, it was Friday past noon and a snowstorm had developed that was sustained over the weekend. The patient continued to do well and was ambulatory Sunday. On Monday morning, the new attendant staff felt that a satisfactory exercise test could obviate the need for invasive management. The patient performed well on this test and was discharged on aspirin, a statin and a beta-blocker plus clopidogrel for 3 months. His life was uneventful a year later.

Fibrinolysis remains a cornerstone for the treatment of ST elevation myocardial infarction (STEMI) when primary percutaneous coronary intervention (PCI) is not readily available. However, important clinical questions arise with fibrinolysis. Should a 'pharmacoinvasive approach', that is, fibrinolysis followed by routine coronary angiography with PCI when anatomically feasible, be performed in patients with STEMI or is an equivalent clinical benefit achieved with a selective, ischemia-guided approach to coronary angiography and revascularization? And if a pharmacoinvasive approach is superior, when after fibrinolysis should patients undergo invasive intervention? And is the magnitude of benefit compelling enough to justify systematic recourse to the limited and costly resources of tertiary cardiac care?

Older studies (≥ 15 years ago) showed no benefit of systematic invasive cardiac management of patient with STEMI after fibrinolysis[1-3]. However, these studies are no longer considered relevant in the 'modern era' because of the increasing sophistication of invasive technology with generalized use of stents, platelet 2b/3a glycoprotein antagonists and thienopyridines (such as clopidogrel)[4,5]. North American guidelines have tended towards a progressive acceptance of routine PCI as part of an invasive strategy after fibrinolytic therapy[6-8]. An even stronger endorsement of the pharmacoinvasive strategy comes from the most recent European guidelines[9] that recommend, even in the case of successful fibrinolysis, coronary angiography within 3-24 hours. Thus, the pharmacoinvasive approach is increasingly becoming the norm. This evolution in clinical practice and in expert opinion and its shades of differences based on a similar body of knowledge has prompted us to perform a systematic review of the evidence from randomized clinical trials (RCTs) examining this issue.

## Methods

This review was performed in accordance with the Preferred Reporting Items for Systematic Reviews and Meta-Analyses, or PRISMA statement[10].

### Data sources

We systematically searched EMBASE and Medline to identify all RCTs published in English that compared systematic invasive management of STEMI patients after fibrinolysis with prevailing 'standard' or 'usual care'. A detailed description of our literature search strategy, conducted in June 2010, and corresponding key words is found in Additional file 1. Briefly, we combined keyword searches for PCI or cardiac catheterization, fibrinolysis or thrombolysis, STEMI or myocardial infarction, and trials. We then restricted our search to clinical trials, human subjects, and English language publications. We also hand-searched references of relevant publications for additional trials.

### Study selection

We included RCTs that compared fibrinolysis followed by standard or usual care with fibrinolysis plus routine invasive management, performed at any time following fibrinolysis. We included studies that differed only in the timing of invasive management after fibrinolysis because later invasive management was their standard of care and because these studies have been invoked in support of a routine pharmacoinvasive approach. We excluded 'rescue PCI' studies (where emergency PCI was performed because fibrinolysis was assessed to have failed)[11-13] and 'facilitated PCI' studies (where emergency PCI was considered part of the original fibrinolytic strategy and where the comparator was primary PCI)[14,15]. In addition, we excluded 12 trials published ≥ 15 years ago as they have been critiqued in the more contemporary era on the grounds that they are no longer relevant because of advances in cardiac percutaneous technology and adjuvant drug treatment[4,5].

### Data extraction

We extracted data regarding study design, including sample size, duration of follow-up, and use of angiography, PCI, and clopidogrel (or ticlopidine) in each treatment group, and clinical outcomes at 30 days, 6 months, and 1 year. Where necessary, relative risks, 95% confidence intervals (CI), and p-values were calculated from presented count data. Data were extracted in duplicate, with disagreements resolved by consensus.

### Data synthesis

The disparate protocols and heterogeneous comparative groups of the included trials precluded a formal meta-analysis. The limited available data also prevented the exploration of sources of heterogeneity via meta-analytic tools such as meta-regression. We have therefore opted to systematically review each individual trial.

## Results

**Figure 1:**
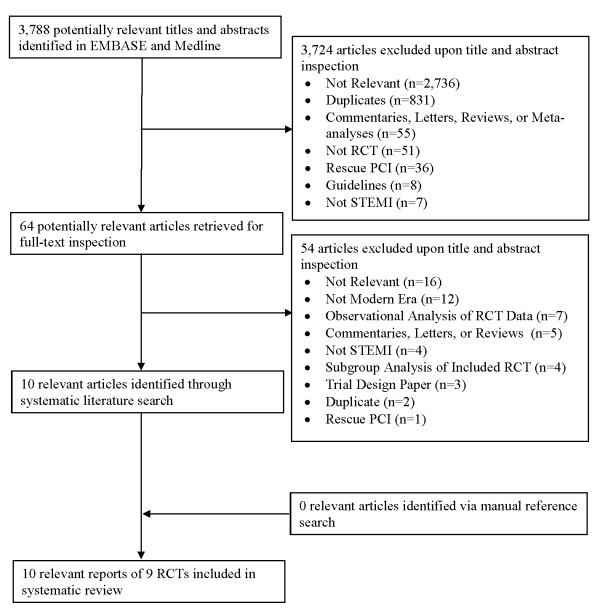
**Flow diagram of literature search of relevant randomized clinical trials (RCTs)**.

Out literature search identified 3,788 potentially relevant publications (Figure 1). Of these publications, 64 were retrieved for full text inspection. A total of 54 articles were excluded upon closer inspection, including 12 trials published ≥ 15 years ago. Consequently, 10 relevant articles of 9 RCTs were included in the present review. These trials randomized a total of 3320 patients.

**Figure 2:**
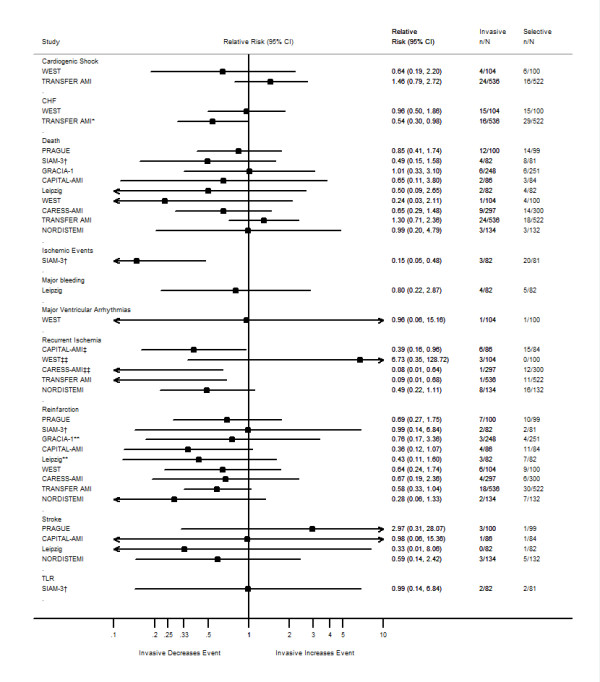
**Thirty-day outcomes of randomized clinical trials comparing an invasive treatment strategy to a delayed invasive or ischemia-guided approach among STEMI patients treated with fibrinolysis**. Abbreviations: CHF = Congestive Heart Failure; TLR = Target Lesion Revascularization. *New or worsening CHF. **Non-fatal reinfarction. ‡ Recurrent unstable ischemia. ‡‡ Refractory ischemia. † Data are based on the 163 patients who met the secondary inclusion criteria rather than the 197 who were randomized. †† Data are based on the 197 patients who were randomized.

**Figure 3:**
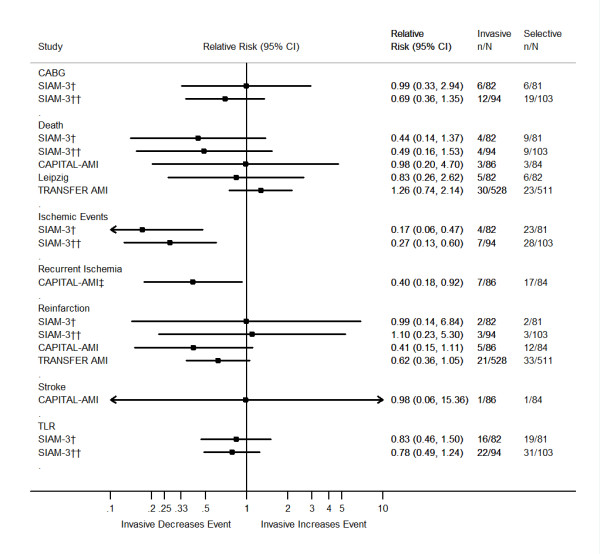
**Six-month outcomes of randomized clinical trials comparing an invasive treatment strategy to a delayed invasive or ischemia-guided approach among STEMI patients treated with fibrinolysis**. Abbreviations: CABG = Coronary Artery Bypass Graft Surgery; TLR = Target Lesion Revascularization. ‡ Recurrent unstable ischemia. † Data are based on the 163 patients who met the secondary inclusion criteria rather than the 197 who were randomized. †† Data are based on the 197 patients who were randomized.

**Figure 4:**
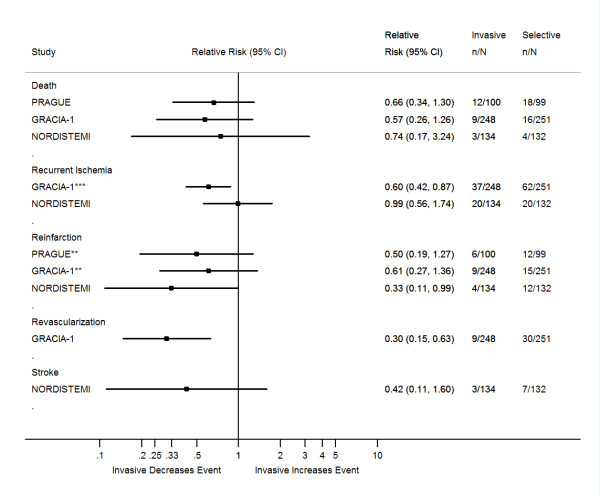
**One-year outcomes of randomized clinical trials comparing an invasive treatment strategy to a delayed invasive or ischemia-guided approach among STEMI patients treated with fibrinolysis**. **Non-fatal reinfarction. ***Readmission due to ischemia.

**Figure 5:**
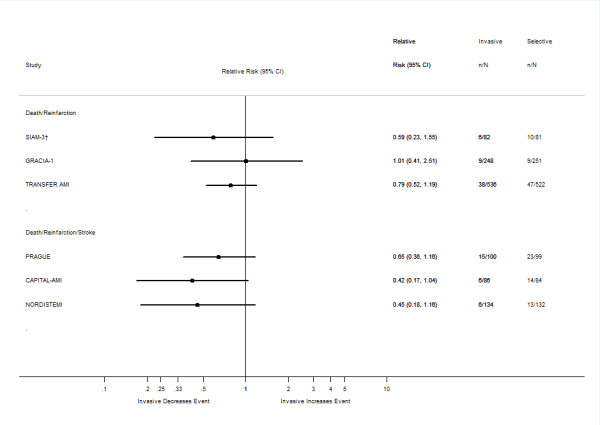
**Thirty-day composite outcomes of death/reinfarction and death/reinfarction/stroke reported in randomized clinical trials comparing an invasive treatment strategy to a delayed invasive or ischemia-guided approach among STEMI patients treated with fibrinolysis**. †Data are based on the 163 patients who met the secondary inclusion criteria rather than the 197 who were randomized.

The characteristics of the trials examined and their outcomes are summarized in Tables 1, 2, 3. We have also created forest plots that sum up the individual outcomes of the trials at 30 days and when available at 6 months and one year (Figures 2, 3, 4). An additional forest plot was created to summarize two 30-day composite endpoints (death/reinfarction and death/reinfarction/stroke) that were reported by three RCTs (Figure 5). Due to the inconsistent reporting of other composite endpoints, it was not practical to present these data graphically. Instead, we summarize these data as part of Tables 2 and 3. The pertinent points of the individual studies are reviewed below.

**Table 1:** Study characteristics of randomized clinical trials comparing an invasive treatment strategy to a delayed invasive or ischemia-guided approach among STEMI patients treated with fibrinolysis.

Study	Year	Sample Size	Comparator	Angiogram in Invasive Group (%)	Angiogram in Selective Group (%)	PCI in Invasive Group (%)	PCI in Selective Group (%)	Clopidogrel Use in Invasive Group (%)	Clopidogrel Use in Selective Group (%)	Follow-Up (Month)
PRAGUE[16,17]	2000	300*	Streptokinase	NR	NR	82^†^	7^†^	All received ticlopidine		1

SIAM-3[18]	2003	197^‡^	Reteplase with elective stenting 2 weeks post-thrombolysis	100	100	100	100	100	100^#^	9.4 ± 7.4

GRACIA-1[19]	2004	500	Recombinant tissue plasminogen activator with ischemia-guided approach	100	21	81.4^¥^	20.3	NR	NR	12

CAPITAL-AMI[20]	2005	170	TNK-alone with rescue PCI PRN	100	67	100	50	91	57	6

Leipzig[23]	2005	164	Combination half-dose reteplase and abciximab with rescue PCI PRN and elective PCI recommended before hospital discharge	100	NR	96	91	88	77^##^	6

WEST[25]	2006	304^€^	TNK followed by 'usual standard of care'	98.1	NR	78.8^£^	60	NR	NR	1

CARESS-AMI[26]	2008	600	Combination half-dose reteplase and abciximab with transfer for rescue PCI PRN^††^	97.0	35.7	85.6	30.3	85.9**	57.1**	1

TRANSFER AMI[27]	2009	1,059	TNK with rescue PCI PRN and recommended angiogram within 2 weeks of Index MI	98.5	89	85	67	89^††^	69^††^	6

NORDISTEMI[28]	2009	266^‡‡^	TNK with ischemia-guided approach	99^¥¥^	95^¥¥^	89	71	100		12

Abbreviations: MI: myocardial infarction; PCI: percutaneous coronary intervention; PRN: as needed; STEMI: ST-elevation myocardial infarction; TNK: tenecteplase. *Of the 300 patients randomized in PRAGUE, 101 were randomized to primary PCI and are not included in this analysis. A total of 100 patients were randomized to streptokinase + PCI and 99 were randomized to streptokinase alone. ^†^These data represent procedures that occurred within the index hospitalization; 5 patients in the invasive group and 11 patients (including 7 rescue PCI) in the conservative group underwent PCI in the 30 days following randomization. ^‡^In the SIAM-3 trial, 197 patients were initially randomized. However, only 163 met their secondary inclusion criteria. The remaining 34 patients were excluded. Data presented here are based on the 163 patients who met the secondary inclusion criteria. ^#^Although all patients in the SIAM study received clopidogrel, patients randomized to the delayed invasive approach received clopidogrel 2 weeks later than those randomized to an early invasive approach. ^¥^Includes 199 patients who underwent stenting of culprit lesion and 3 who underwent stenting of non-culprit lesions. ^€^In the WEST study, 304 patients were randomized to 3 treatment arms. However, only 2 of these arms are considered here (n = 204). ^##^Most patients in the selective group received clopidogrel at the time of PCI several days after the index event. ^£^Includes 1 patient revascularized after index hospitalization but within 30 days of the index event. **Denotes clopidogrel use at discharge. ^††^Denotes clopidogrel use before admission or within the first 6 hours. ^‡‡^A total of 266 patients were randomized. However, 4/138 randomized to immediate transfer for PCI and 6/138 randomized to the ischemia-guided approach were excluded following randomization. Consequently, all analyses are based on 134 and 132 patients, respectively. ^¥¥ ^In the early invasive group, 83% underwent angiography within 3 hours of TNK and 99% with 12 hours. In the selective group, 12% underwent angiography with 3 hours, 33% within 12 hours, and 86% within 30 days.

**Table 2:** Thirty-day outcomes of randomized clinical trials comparing an invasive treatment strategy to a delayed invasive or ischemia-guided approach among STEMI patients treated with fibrinolysis.

Study	Outcome	Risk in Invasive Group (n/N)	Risk in Selective Group (n/N)	Relative Risk (95% CI)	p-value
PRAGUE[16]	Death/Reinfarction/Stroke*	15/100	23/99	0.65 (0.36, 1.16)	0.14^‡^

	Death	12/100	14/99	0.85 (0.41, 1.74)	0.66^‡^

	Reinfarction	7/100	10/99	0.69 (0.27, 1.75)	0.43^‡^

	Stroke	3/100	1/99	2.97 (0.31, 28.1)	0.62^‡^

SIAM-3[18]^†^	CABG	0/82	0/81	-	1.00

	TLR	2/82	2/81	0.99 (0.14, 6.84)^‡^	0.685

	Ischemic Events	3/82	20/81	0.15 (0.05, 0.48)^‡^	0.01

	Reinfarction	2/82	2/81	0.99 (0.14, 6.84)^‡^	0.685

	Death	4/82	8/81	0.49 (0.15, 1.58)^‡^	0.179

	Death/Reinfarction	6/82	10/81	0.59 (0.23, 1.55)^‡^	0.208

	Death/Reinfarction/TLR	6/82	11/81	0.54 (0.21, 1.39)^‡^	0.146

	Death/Reinfarction/TLR/Ischemic Events	7/82	25/81	0.28 (0.13, 0.60)^‡^	0.001

GRACIA-1[19]	Death	6/248	6/251	1.01 (0.33, 3.10)	0.84

	Non-Fatal Reinfarction	3/248	4/251	0.76 (0.17, 3.36)	0.98

	Death/Non-Fatal Reinfarction	9/248	9/251	1.01 (0.41, 2.51)	0.97

	Death/Non-Fatal Reinfarction/Revascularization	12/248	16/251	0.76 (0.37, 1.57)	0.46

CAPITAL-AMI[20]	Death/Recurrent MI/Recurrent Unstable Ischemia, Stroke	8/86	18/84	0.43 (0.20, 0.93)	0.03

	Death	2/86	3/84	0.64 (0.11, 3.75)	0.68

	Reinfarction	4/86	11/84	0.35 (0.12, 1.06)	0.06

	Recurrent Unstable Ischemia	6/86	15/84	0.39 (0.16, 0.95)	0.04

	Stroke	1/86	1/84	0.97 (0.61, 15.18)	1.00

	Death/Reinfarction/Stroke	6/86	14/84	0.41 (0.17, 1.03)	0.06

Leipzig[23]	Death	2/82	4/82	0.50 (0.09, 2.65)^‡^	0.68^‡^

	Non-Fatal Reinfarction	3/82	7/82	0.43 (0.11, 1.60)^‡^	0.33^‡^

	Stroke	0/82	1/82	-	1.00^‡^

	Major bleeding	4/82	5/82	0.80 (0.22, 2.87)^‡^	1.00^‡^

	Death/Reinfarction/Stroke/Major Bleeding	9/82	17/82	0.52 (0.23, 1.18)	0.13^¥^

WEST[25]	Death/Reinfarction/Refractory Ischemia/CHF/Cardiogenic Shock/Major Ventricular Arrhythmia*	25/104	25/100	0.96 (0.59, 1.56) ^‡^	0.87^‡^

	Death	1/104	4/100	0.24 (0.03, 2.11) ^‡^	0.21^‡^

	Reinfarction	6/104	9/100	0.64 (0.24, 1.74) ^‡^	0.38^‡^

	CHF	15/104	15/100	0.96 (0.50, 1.86)^‡^	0.91^‡^

	Cardiogenic Shock	4/104	6/100	0.64 (0.19, 2.20)^‡^	0.53^‡^

	Refractory Ischemia	3/104	0/100	-	0.25^‡^

	Major Ventricular Arrhythmias	1/104	1/100	0.96 (0.06, 15.2)^‡^	1.00^‡^

CARESS-AMI[26]	Death/Reinfarction/Refractory Ischemia*	13/297	32/300	0.41 (0.22, 0.77)^‡^	0.005

	Death	9/297	14/300	0.65 (0.29, 1.48)^‡^	0.40

	Reinfarction	4/297	6/300	0.67 (0.19, 2.36)^‡^	0.75

	Refractory Ischemia	1/297	12/300	0.08 (0.01, 0.64)^‡^	0.003

TRANSFER AMI[27]	Death/Reinfarction/Recurrent Ischemia/New or Worsening CHF/Cardiogenic Shock*	59/536	90/522	0.64 (0.47, 0.87)	0.004

	Death	24/536	18/522	1.30 (0.71, 2.36)	0.39

	Reinfarction	18/536	30/522	0.57 (0.33, 1.04)	0.06

	Death/Reinfarction	38/536	47/522	0.79 (0.52, 1.19)	0.25

	Recurrent Ischemia	1/536	11/522	0.09 (0.01, 0.68)	0.003

	Death/Reinfarction/Recurrent Ischemia	39/536	58/522	0.65 (0.44, 0.96)	0.03

	New or Worsening CHF	16/536	29/522	0.54 (0.30, 0.98)	0.04

	Cardiogenic Shock	24/536	16/522	1.46 (0.79, 2.72)	0.23

NORDISTEMI[28]	Death/Reinfarction/Stroke/New Ischemia	14/134	28/132	0.49 (0.27, 0.89)^‡^	0.02^‡^

	Death/Reinfarction/Stroke	6/134	13/132	0.45 (0.18, 1.16)^‡^	0.09^‡^

	Death	3/134	3/132	0.99 (0.20, 4.79)^‡^	1.00^‡^

	Reinfarction	2/134	7/132	0.28 (0.06, 1.33)^‡^	0.10^‡^

	Stroke	3/134	5/132	0.59 (0.14, 2.42)^‡^	0.50^‡^

	Recurrent Ischemia	8/134	16/132	0.49 (0.22, 1.11)^‡^	0.08^‡^

Abbreviations: CHF: congestive heart failure; CI: confidence interval; MI: myocardial infarction; TLR: target lesion revascularization.

*Denotes primary endpoint of the trial. † Data are based on the 163 patients who met the secondary inclusion criteria rather than the 197 who were randomized. ^†† ^Data are based on the 197 patients who were randomized. ‡ Calculated using data presented in the original manuscript. ^¥ ^Denotes p-value from log-rank test.

**Table 3:** Six-and 12-month outcomes of randomized clinical trials comparing an invasive treatment strategy to a delayed invasive or ischemia-guided approach among STEMI patients treated with fibrinolysis.

Study	Follow-Up	Outcome	Risk in Invasive Group (n/N)	Risk in Selective Group (n/N)	Relative Risk (95% CI)	p-value
PRAGUE[17]	12 Months	Death/Non-Fatal Reinfarction	18/100	30/99	0.59 (0.36, 0.99)	0.04^‡^

		Death	12/100	18/99	0.66 (0.34, 1.30)	0.22^‡^

		Non-Fatal Reinfarction	6/100	12/99	0.50 (0.19, 1.27)	0.13^‡^

SIAM-3[18]	6 Months^†^	CABG	6/82	6/81	0.99 (0.33, 2.94)^‡^	0.609

		TLR	16/82	19/81	0.83 (0.46, 1.50)^‡^	0.336

		Ischemic Events	4/82	23/81	0.17 (0.06, 0.47)^‡^	0.001

		Reinfarction	2/82	2/81	0.99 (0.14, 6.84)^‡^	0.685

		Death	4/82	9/81	0.44 (0.14, 1.37)^‡^	0.119

		Death/Reinfarction	6/82	11/81	0.54 (0.21, 1.39)^‡^	0.146

		Death/Reinfarction/TLR	20/82	29/81	0.68 (0.42, 1.10)^‡^	0.078

		Death/Reinfarction/TLR/Ischemic Events*	21/82	41/81	0.51 (0.33, 0.78)^‡^	0.001

	6 Months^††^	CABG	12/94	19/103	0.69 (0.36, 1.35)^‡^	0.185

		TLR	22/94	31/103	0.78 (0.49, 1.24)^‡^	0.185

		Ischemic Events	7/94	28/103	0.27 (0.13, 0.60)^‡^	0.001

		Reinfarction	3/94	3/103	1.10 (0.23, 5.30)^‡^	0.614

		Death	4/94	9/103	0.49 (0.16, 1.53)^‡^	0.164

		Death/Reinfarction	7/94	12/103	0.64 (0.27, 1.56)^‡^	0.225

		Death/Reinfarction/TLR	26/94	41/103	0.69 (0.46, 1.04)^‡^	0.049

		Death/Reinfarction/TLR/Ischemic Events	28/94	55/103	0.56 (0.39, 0.80)^‡^	0.001

GRACIA-1[19]	12 Months	Death	9/248	16/251	0.55 (0.22, 1.36)	0.16

		Non-Fatal Reinfarction	9/248	15/251	0.60 (0.27, 1.36)	0.22

		Death/Non-Fatal Reinfarction	17/248	29/251	0.59 (0.33, 1.05)	0.07

		Revascularization	9/248	30/251	0.30 (0.15, 0.62)	0.001

		Readmission due to Ischemia	37/248	62/251	0.60 (0.42, 0.87)	0.006

		Death/Non-Fatal Reinfarction/Revascularization*	23/248	51/251	0.44 (0.28, 0.70)	0.0008

CAPITAL-AMI[20]	6 Months	Death/Recurrent MI/Recurrent Unstable Ischemia, Stroke*	10/86	20/84	0.48 (0.24, 0.96)	0.04

		Death	3/86	3/84	0.95 (0.20, 4.59)	1.00

		Reinfarction	5/86	12/84	0.40 (0.15, 1.08)	0.07

		Recurrent Unstable Ischemia	7/86	17/84	0.39 (0.17, 0.90)	0.03

		Stroke	1/86	1/84	0.95 (0.60, 14.99)	1.00

		Death/Reinfarction/Stroke	8/86	15/84	0.51 (0.23, 1.14)	0.12

Leipzig[23]	6 Months	Death	5/82	6/82	0.83 (0.22, 2.99)	0.68^¥^

		Death/Reinfarction/Stroke/Major Bleeding	12/82	21/82	0.57 (0.28, 1.13)	0.10^¥^

TRANSFER AMI[27]	6 Months	Death	30/528	23/511	1.27 (0.77, 2.23)	0.39

		Reinfarction	21/528	33/511	0.60 (0.34, 1.05)	0.07

		Death/Reinfarction	47/528	54/511	0.83 (0.55, 1.25)	0.36

NORDISTEMI[28]	12 Months	Death/Reinfarction/Stroke/New Ischemia*	28/134	36/132	0.77 (0.50, 1.18)^‡^	0.22^‡^

		Death/Reinfarction/Stroke	8/134	21/132	0.38 (0.17, 0.82)^‡^	0.008^‡^

		Death	3/134	4/132	0.74 (0.17, 3.24)^‡^	0.72^‡^

		Reinfarction	4/134	12/132	0.33 (0.11, 0.99)^‡^	0.04^‡^

		Stroke	3/134	7/132	0.42 (0.11, 1.60)^‡^	0.22^‡^

		Recurrent Ischemia	20/134	20/132	0.99 (0.56, 1.74)^‡^	0.96^‡^

Abbreviations: CHF: congestive heart failure; CI: confidence interval; MI: myocardial infarction; TLR: target lesion revascularization. * Denotes primary endpoint of the trial. † Data are based on the 163 patients who met the secondary inclusion criteria rather than the 197 who were randomized. ^†† ^Data are based on the 197 patients who were randomized. ‡ Calculated using data presented in the original manuscript. ^¥ ^Denotes p-value from log-rank test.

### PRAGUE Study

In this trial, 99 patients with STEMI were randomized in non-PCI hospitals to streptokinase alone and 100 patients were randomized to streptokinase followed by systematic transfer for immediate PCI[16]. Revascularization was performed within 30 days in 14% (including rescue PCI in 7%) of the streptokinase alone group. The occurrences of death, reinfarction, and stroke at 30 days were 14%, 10%, and 1%, respectively, in the streptokinase alone group versus 12%, 7%, and 3%, respectively, in the streptokinase plus immediate PCI group. The relative risk for the occurrence of the composite endpoint of death, reinfarction, and stroke in favor of the latter group was 0.65 (95% CI: 0.36-1.16, p = 0.14). The authors later reported outcomes at one year[17]. The composite endpoint of death, reinfarction, and stroke was not reported at one year. Rather the endpoint of death and reinfarction was reported as 30% in the streptokinase alone group versus 18% in the pharmacoinvasive group (relative risk 0.59; 95% CI: 0.36-0.99; p = 0.04). Limitations of this study are the use of a fibrinolytic strategy generally considered to be inferior, the relatively small number of patients, and the lack of consistency in reporting the same endpoint at the 2 points in time.

### SIAM-3

In this study, 197 STEMI patients were treated with reteplase and randomized to 1 of 2 approaches: immediate stenting (median time, 3.5 hours) or elective stenting after 2 weeks[18]. Thirty-four patients were excluded after randomization because of the need for bypass surgery or the presence of a non-significant or unapparent infarct-related culprit lesion. Immediate stenting resulted in a reduction in the primary 6-month composite endpoint of death, reinfarction, target lesion revascularization, and recurrent ischemic events (25.6% versus 50.6%; relative risk 0.51; 95% CI: 0.33-0.78; p = 0.001). The difference between the 2 groups was driven by ischemic events (4.9% vs. 28.4%, relative risk 0.17; 95% CI: 0.06-0.47; p = 0.001), more than by death or reinfarction (7.3% vs. 13.6%; relative risk 0.54; 95% CI; 0.21-1.39; p = 0.146). Furthermore, this relatively small study did not examine the role of a selective invasive approach as all patients received the invasive intervention, merely at 2 different times (3.5 hours versus 2 weeks). The pertinence of tallying ischemic events that occurred after 2 weeks and out to 6 months (at least 50% by visual inspection of the Kaplan Meier survival analysis) is intuitively unclear since by 2 weeks both arms had received the same treatment. The large exclusion of patients following randomization is also a threat to the internal validity of the study, as is the unaccounted role of differential exposure to clopidogrel between the two groups (given within a few hours in one group but 2 weeks later in the other group).

### GRACIA-1

The GRACIA-1 study randomized 500 STEMI patients to a routine invasive strategy within 24 hours of fibrinolysis or to an ischemia-guided 'conservative' approach[19]. The hypothesis of this study was well formulated, its design clear, and its sample size relatively robust. Coronary angiography was performed at a median of 17 hours after fibrinolysis in the invasive group. In-hospital coronary angiography was performed in 21% of the ischemia-guided group. Criteria for recourse to coronary angiography and revascularization in the ischemia-guided group were clearly defined (spontaneous recurrent ischemia with ECG changes or evidence on non-invasive stress testing of a severe ischemic substrate). Thus, in this study, there was a clear distinction between the 2 clinical approaches conducive for a meaningful comparison. Revascularization (with stenting in over 95% of patients) was performed in 83% of the invasive group compared with 20% of the ischemia-guided group. It was not specified whether any rescue PCI was performed. The primary combined endpoint of death, reinfarction, and revascularization at 12 months occurred in 21% of the ischemia-guided group versus 9% of the invasive group (relative risk: 0.44, 95% CI: 0.28-0.70; p = 0.0008). This difference was essentially driven by differences in revascularization procedures between groups. However, the inclusion of this component of the composite endpoint is problematic as it involves the actual intervention in only one group. Thus, the inference that this study supports the use of a systematic invasive approach following fibrinolysis is valid if one accepts the authors' position of not counting the initial 208 in-hospital revascularizations performed in the invasive group. If these initial procedures are counted in the 12-month total of revascularization procedures, an entirely different conclusion is drawn, with more than twice as many revascularizations performed in the invasive group compared with the ischemia-guided group. The unbalanced use of clopidogrel is again a confounding variable in this study. Although it is unspecified, it can be assumed that nearly all patients in the invasive group were treated for some time with clopidogrel (or ticlopidine) while it is likely that few patients in the ischemia-guided group received this treatment prior to any revascularization procedure. As well, there was a lower enzyme threshold (3 times the normal value of creatine kinase MB isoenzyme) for defining reinfarction within 48 h of fibrinolysis compared with the definition of reinfarction within 48 h of invasive intervention (5 times the normal value), which may bias results against the conservative strategy. Nevertheless, there was no statistically significant difference at one year in death or re-infarction between the 2 groups arms (risk ratio: 0.59; 95% CI: 0.33-1.05; p = 0.07) but our ability to draw any meaningful causal inferences is limited by these potential biases.

### CAPITAL-AMI

In this study, 170 STEMI patients treated with fibrinolysis were randomized to routine immediate PCI or fibrinolysis alone with rescue or deferred PCI as clinically indicated[20]. Cardiac catheterization was performed in-hospital in 67% of the patients in the fibrinolysis-alone group, with 50% of patients in this group undergoing PCI (including 14% rescue PCI). The primary composite endpoint of death, reinfarction, recurrent unstable ischemia, or stroke at 6 months was reduced with the pharmacoinvasive strategy from 24.4% to 11.6% (relative risk: 0.48; 95% CI: 0.24-0.96; p = 0.04). This difference was driven by a reduction in the rate of recurrent unstable ischemia that included reinfarction (20.7% vs. 8.1%; relative risk 0.39; 95% CI: 0.17-0.90; p = 0.03). There was no difference in death, stroke, heart failure/cardiogenic shock, left ventricular ejection fraction, or treadmill exercise duration between the 2 groups. Interestingly, there was also no improvement in ST-segment resolution, an important predictor of mortality[21], with routine immediate PCI[22]. Again in this relatively small study, unbalanced use of clopidogrel (91% in the pharmacoinvasive arm compared with 57% in the fibrinolysis-alone arm) may well have confounded the difference in recurrent unstable ischemia between the 2 approaches.

### LEIPZIG Study

This RCT compared immediate versus delayed PCI in 164 STEMI patients treated with fibrinolysis (half-dose reteplase) plus abciximab[23]. The composite secondary clinical endpoint of death, reinfarction, major bleeding, and stroke at 6 months was not significantly reduced with immediate PCI (15% versus 25%; relative risk: 0.57; 95% CI: 0.28-1.13; p = 0.1), although the study did have limited power to attain this endpoint. As with SIAM-3, the Leipzig study examined the timing of PCI rather than the benefits of a pharmacoinvasive versus ischemia-guided approach. Timing of the introduction of clopidogrel was not specified but likely was postponed in the delayed PCI group, potentially confounding the assessment of outcome in the 2 groups. Finally, the combination of half-dose fibrinolysis and platelet glycoprotein inhibition has not been found to be more efficacious than standard fibrinolysis and also results in more bleeding complications, particularly in older patients[24]. Thus, the findings of this small study are not readily pertinent to an examination of the benefit of a pharmacoinvasive approach versus a selective invasive approach based on risk and clinical evolution in patients with STEMI.

### WEST study

This trial randomized 304 patients with STEMI to fibrinolysis alone (n = 100), fibrinolysis plus transfer for PCI within 24 hours (n = 104), or primary PCI (n = 100)[25]. In-hospital revascularization was undertaken in 60% of the fibrinolysis-alone patients (rescue PCI in 14%). There was no significant difference in the 30-day primary composite efficacy endpoint (death, reinfarction, heart failure, cardiogenic shock, refractory ischemia, and major ventricular arrhythmias) that occurred in 24% of the pharmacoinvasive group and in 25% of the fibrinolysis alone group (relative risk 0.96; 95% CI: 0.59-1.56; p = 0.87). A reduced composite endpoint of death and reinfarction was not significant between the two groups (6.7% in the pharmacoinvasive versus 13.0% in the fibrinolysis alone group), although the power to detect differences was limited in this small study. No information was available about the nature and timing of the small number of reinfarctions but systematic use of clopidogrel in the pharmacoinvasive group and its likely less frequent use in the fibrinolysis group before any PCI as well as a higher threshold for defining myocardial infarction if it was related to PCI are factors that likely attenuate the small difference in reinfarction noted in the 2 groups.

### CARESS-AMI

This study randomized 600 patients, aged ≤75 years, to a pharmacoinvasive approach or standard ischemia-guided management (including rescue PCI) after receiving half-dose reteplase and abciximab[26]. In the latter group, in-hospital angiography was performed in 36% and PCI in 30%. The primary outcome was a composite endpoint of death, reinfarction, and refractory ischemia at 30 days and occurred in 13 patients (4.4%) in the pharmacoinvasive group versus 32 patients (10.7%) in the standard care/rescue group (relative risk 0.41; 95% CI 0.22-0.77; p = 0.005). This endpoint was driven by reduced refractory ischemia that occurred at 30 days in only one patient (0.3%) in the pharmacoinvasive group versus 12 patients (4.3%) in the standard care/rescue group (p = 0.003). The corresponding occurrences of death and reinfarction at 30 days were 3.0% vs. 4.7% (relative risk 0.65; 95% CI: 0.29-1.48; p = 0.4) and 1.3% vs. 2.0% (relative risk 0.68; 95% CI: 0.19-2.36; p = 0.75), respectively. Clear and equal application of diagnostic tests to both groups following randomization was not specified, leaving the possibility of a diagnostic bias for ischemia detection in a necessarily unblinded study.

### TRANSFER AMI

In this study, 528 patients were randomized to PCI performed within 4 hours after fibrinolysis (early pharmacoinvasive group) and 511 patients were randomized to 'standard' treatment (defined as rescue PCI performed for clinically failed reperfusion and a recommendation that cardiac catheterization be performed in all patients within 2 weeks)[27]. The use of cardiac catheterization in the standard treatment group was consequently very high (89%). This was therefore essentially a trial of the timing of PCI after fibrinolysis that compared an early to a later pharmacoinvasive approach. The composite endpoint of 30-day death, reinfarction, congestive heart failure, severe recurrent ischemia, and shock occurred in 11.0% of the immediate PCI arm and 17.2% of the standard arm (relative risk 0.64; 95% CI: 0.47-0.87; p = 0.004). The difference in the 2 groups was again driven by recurrent ischemia (0.2% vs. 2.1%; relative risk 0.09; 95% CI: 0.01-0.68; p = 0.003) and reinfarction (3.4% vs. 5.7%; relative risk 0.57; 95% CI: 0.33-1.04; p = 0.06). At 6 months, there was no significant difference in death or reinfarction between the 2 groups (8.9% in the routine early group vs 10.6% in the standard group; relative risk 0.83; 95% CI: 0.55-1.25; p = 0.36). Again, the use of clopidogrel was not equal in the 2 arms; although relatively high in the standard treatment arm (69% within the first 6 h), it was lower than in the routine early PCI group (89%).

### NORDISTEMI

This RCT compared a strategy of immediate transfer for PCI with an ischemia-guided approach after fibrinolysis (pre-hospital in 57%) in 266 patients situated too far away for timely primary PCI[28]. In the immediate transfer group, the median time from receiving fibrinolysis to arrival at the catheterization laboratory was 130 min and PCI was performed in 89%. In the ischemia-guided management arm, 27% underwent rescue PCI and 93% of the remainder had cardiac catheterization in the following days (at a median time of 5.5 days for the group as a whole). PCI was performed in 71% and coronary bypass surgery in 12% of this 'conservative' group. The terms 'ischemia-guided management' and 'conservative' used by the authors to characterize this latter group are somewhat misnomers because nearly all these patients underwent invasive management sooner or later. The primary endpoint of death, reinfarction, stroke, and recurrent ischemia at 12 months was not significantly different between the 2 strategies (21% in early invasive group vs. 27% in 'conservative group'; relative risk: 0.77; 95% CI: 0.50-1.18; p = 0.22). The authors highlight the finding that an endpoint restricted to death, stroke, and reinfarction at 12 months was significantly reduced in the immediate pharmacoinvasive group (6% vs 16%, relative risk: 0.38; 95% CI: 0.17-0.82; p = 0.008). The pertinence of this finding is difficult to gauge because between 30 days and 12 months, there was a marked increase in recurrent ischemia in the immediate pharmacoinvasive group while in the same period, there was an increase in stroke and reinfarction in the 'conservative' arm. Beyond the play of chance, it is unclear why these directionally discordant vascular event rates should have occurred within this later time frame.

## Discussion

This complete and detailed review of the RCTs that evaluated routine invasive management against a comparator strategy following fibrinolysis in patients with STEMI has found important methodological limitations to performing a meta-analysis of the available evidence. Importantly, the comparator to routine invasive management has varied widely from systematic but deferred invasive management to large differences in the proportion of patients in the comparator arm who underwent invasive management. Additionally, several potential biases were identified that might confound the findings of the individual RCTs. Thus, this systematic review concludes that definitive RCT evidence in favor of routine invasive management following fibrinolysis in patients with STEMI is presently lacking.

Although primary PCI has tended to become the favored reperfusion therapy wherever it is readily available, a substantial proportion of patients with STEMI still receive fibrinolysis. Since the widespread introduction of fibrinolysis 3 decades ago, a recurring clinical question has been: Should all patients be subsequently routinely referred for invasive management (coronary angiography and anatomically driven revascularization)? Or, following fibrinolysis, should invasive management be more selective or 'ischemia-guided', based on higher risk characteristics such as recurrent or refractory ischemia and non-invasive risk stratification such as an exercise test or myocardial imaging suggesting the presence of a significant ischemic substrate? A secondary question for clinicians favoring routine invasive intervention after fibrinolysis has been its timing: earlier or later? The issue at stake is important both clinically and from the perspective of allocation of costly technological infrastructure. Should treatment of STEMI systematically engage the resources of tertiary cardiac care or can clinicians exercise sufficient clinical acumen to selectively reserve invasive management to identifiable high-risk patients without prejudice to overall patient welfare? Guidelines have attempted to address these questions but the strength of evidence on which these recommendations are based has not been critically reviewed.

In the more contemporary era, fueled by post-hoc analysis of clinical trials[29,30] and interpretation of registry findings[31] and RCTs examining either this question or the secondary question of the timing of invasive management, patients are increasingly routinely referred, and ever earlier, for invasive management after fibrinolysis. A meta-analysis by Wijeysundera et al. examined 5 contemporary studies purporting to compare a pharmacoinvasive approach versus 'ischemia-guided management'[32]. In the present review, we have added 4 additional RCTs, 2 that used a combination of a fibrinolytic and a platelet 2b/3a antagonist[23,26] plus 2 other studies published since this meta-analysis[27,28]. Like the meta-analysis by Wijeysundera et al., all 9 RCTs suggest a benefit, albeit relatively small in absolute terms, from a strategy of routine and ever earlier invasive management following fibrinolysis. Consequently, a similar benefit was found in 2 very recent meta-analyses both of which compared early/immediate routine invasive management with 'standard therapy'[33] or 'a more conservative strategy'[34] that was without distinction either deferred routine invasive management or an ischemia-guided strategy.

However, given the diverse and non-standardized study interventions, routine invasive versus a fluctuating standard care approach (with invasive rates varying from 7% to 67%) or simply an early versus deferred universal invasive strategy (in which there was no contrast in intervention rates only in timing), we believe a quantitative meta-analysis is inappropriate. We have therefore chosen instead to qualitatively review the RCTs individually. Other difficulties in determining if these RCTs establish a causal benefit of a pharmacoinvasive approach include: use of a sub-optimal fibrinolytic agent[16]; confounding due to the asymmetric use of a second anti-platelet agent (usually clopidogrel) known to significantly reduce recurrent ischemia and reinfarction[18-20,25,27]; preponderance of the 'soft' outcome of recurrent ischemia in the combined primary endpoint in unblinded studies exposing to potential ascertainment bias [18-20,26,27]; information bias when only the number of follow-up invasive procedures are tallied and those occurring early as part of the study design in the pharmacoinvasive arm are not counted[19]; and misclassification bias whereby, the diagnosis of myocardial infarction differs between the invasive and standard arms (generally, a higher cardiac enzyme threshold required for recurrent myocardial infarction following PCI, although the latter does appear to have a better prognosis than a spontaneously occurring myocardial infarction[35]).

Consequently, while these RCTs are regularly cited to support a pharmacoinvasive approach, this systematic review suggests that these potential biases and heterogeneity in the evidence makes definitive conclusions hazardous. Importantly, meta-analysis with a summary effect size and corresponding 95% CI represents only the random error and not the systematic errors associated with these potential biases.

### Is there an optimal rate of invasive management following fibrinolysis?

The RCTs have evaluated routine versus selective invasive management with a wide variation in the latter, or have compared only the timing of a pharmacoinvasive approach. Because of this evidence base and because clinicians increasingly favor the pharmacoinvasive approach, the optimal rate of coronary angiography and revascularization following fibrinolysis in STEMI patients remains unclear. This question assumes that clinical judgment can be sufficiently reliable that the patients who will benefit from invasive management will be accurately identified and unnecessary procedures can be avoided in those who will not derive benefit. Observations from registries and clinical studies do suggest the presence of a threshold effect[36-39]. Above a certain rate of invasive recourse, little or no additional benefit is noted. Below this rate, outcomes analysis suggests suboptimal treatment, placing patients at risk. A systematic review in 2001 concluded that rates of coronary angiography and revascularization following myocardial infarction in excess of 30% and 20%, respectively, might not confer additional benefit in preventing death and reinfarction[40]. Consistent with this, more recently, a substudy of the multinational fibrinolytic GUSTO-V trial examined the relationship between early revascularization (within 7 days of STEMI) and one-year mortality in 13,451 patients. These data suggested an optimal rate of early revascularization of 20-30%[38]. In the studies included in the present review, only GRACIA-1 and CARESS-AMI had rates of angiography/PCI within this range in their ischemia-guided arms[19,26]. This evidence base is clearly insufficient to conclude whether a systematic pharmacoinvasive strategy is superior to an appropriately selective ischemia-guided approach.

## Conclusion

Ever earlier routine coronary angiography and anatomy-driven PCI in STEMI patients treated with fibrinolysis is being strongly promoted by reviews, meta-analyses, supporting editorials, opinion-leaders, and guidelines[5,9,31-34,41-45]. Given these numerous publications, it is not surprising that this aggressive approach to clinical management of STEMI patients has become the *zeitgeist *of acute coronary care. Yet our critical and systematic review of available data from RCTs suggests that evidence to support this 'pharmacoinvasive' approach versus a truly selective ischemia-guided approach is more byzantine than conclusive. We believe guidelines should reflect this uncertainty rather than endorse the prevailing penchant[46]. While awaiting definitive evidence regarding which strategy is superior, the more attractive challenge may lie in not treating all patients in the same aggressive manner but in matching clinical management to the unique profiles of individual patients, their baseline risks, and the specific dynamics of their clinical evolution. Thus, it seems reasonable that following fibrinolysis, patients who evolve well and do not show low-threshold or important ischemia on non-invasive risk stratification like an exercise test can be considered at low risk and can be managed conservatively while higher-risk patients can be treated more aggressively. This approach is at the same time consistent with rational use of expensive resources and ultimately the only sensible way to contain runaway healthcare costs in acute coronary disease without adversely affecting patient outcomes.

## Declaration of Competing interests

The authors declare that they have no competing interests.

## Authors' contributions

PB conceived the project and is the principal author of the manuscript. KBF made substantial contributions to analysis and interpretation of data and critical revision of the manuscript. JB made substantial contributions to interpretation of data and critical revision of the manuscript. All authors have given final approval of the version to be published and take public responsibility for its content.

## Pre-publication history

The pre-publication history for this paper can be accessed here:

http://www.biomedcentral.com/1471-2261/11/34/prepub

## Supplementary Material

Additional file 1 **Appendix**. Literature Search Strategy Click here for file
