# Supplementary material for: Routine invasive management after fibrinolysis in patients with ST-elevation myocardial infarction: a systematic review of randomized clinical trials
Source: BMC Cardiovasc Disord. 2011 Jun 20;11:34. doi: 10.1186/1471-2261-11-34 (PMC3145591; doi:10.1186/1471-2261-11-34)
Supplement: Additional file 1 — Appendix. Literature Search Strategy [file 1471-2261-11-34-S1.DOC]

***Appendix*** – Description of Literature Search Strategy

| Search  Number | Search Terms | Number of  Citations |
| --- | --- | --- |
|  |  |  |
| 1 | PCI OR PTCA OR cardiac catheterization OR angiogram OR angiography OR stent$ OR angioplast$ OR revasculari$ OR percutaneous coronary intervention | 489,945 |
| 2 | fibrinoly$ OR thromboly$ | 121,553 |
| 3 | STEMI OR myocardial infarction$ OR MI | 266,541 |
| 4 | trial OR randomi$ | 1,559,041 |
| 5 | #1 AND #2 AND #3 | 13,827 |
| 6 | #4 and #5 | 5,118 |
| 7 | Limit #6 to (clinical trial, all or clinical trial, phase i or clinical trial, phase ii or clinical trial, phase iii or clinical trial, phase iv or clinical trial or controlled clinical trial or randomized controlled trial) | 4,185 |
| 8 | Limit #7 to Humans | 4,162 |
| 9 | Limit #8 to English Language | 3,788 |
|  |  |  |

Databases Searched: EMBASE (1980 – Week 25 2010), Medline (1950 – June Week 3, 2010)

Date of literature search: June 28^th^, 2010
